# Single-step processing of copper-doped titania nanomaterials in a flame aerosol reactor

**DOI:** 10.1186/1556-276X-6-441

**Published:** 2011-07-06

**Authors:** Manoranjan Sahu, Pratim Biswas

**Affiliations:** 1 Aerosol and Air Quality Research Laboratory, Department of Energy, Environmental and Chemical Engineering, Washington University in St. Louis, St. Louis, MO 63130, USA

## Abstract

Synthesis and characterization of long wavelength visible-light absorption Cu-doped TiO_2 _nanomaterials with well-controlled properties such as size, composition, morphology, and crystal phase have been demonstrated in a single-step flame aerosol reactor. This has been feasible by a detailed understanding of the formation and growth of nanoparticles in the high-temperature flame region. The important process parameters controlled were: molar feed ratios of precursors, temperature, and residence time in the high-temperature flame region. The ability to vary the crystal phase of the doped nanomaterials while keeping the primary particle size constant has been demonstrated. Results indicate that increasing the copper dopant concentration promotes an anatase to rutile phase transformation, decreased crystalline nature and primary particle size, and better suspension stability. Annealing the Cu-doped TiO_2 _nanoparticles increased the crystalline nature and changed the morphology from spherical to hexagonal structure. Measurements indicate a band gap narrowing by 0.8 eV (2.51 eV) was achieved at 15-wt.% copper dopant concentration compared to pristine TiO_2 _(3.31 eV) synthesized under the same flame conditions. The change in the crystal phase, size, and band gap is attributed to replacement of titanium atoms by copper atoms in the TiO_2 _crystal.

## Introduction

Nanosized TiO_2 _has been widely used because of its stability in aqueous environments and low production cost. However, its light absorption range is limited to the ultraviolet (UV) spectrum of light due to its wide band gap (approximately 3.2 eV). To shift the absorption range to the visible spectrum, various approaches have been pursued in the past involving size optimization [1], compositional variation to make sub-oxides [2], surface modification [3], and doping [4-6] to modify the TiO_2 _structure. Among these methods, tailoring the band structures by incorporating a dopant into the host nanomaterial is a promising approach [6-8]. Several studies have reported enhancement of absorbtion in the visible range and photocatalytic activity on doping TiO_2 _by transition metal ions like Cu, Co, V, Fe, Nb, and non-metal like N, S, F [4,5,9-11]. However, a major challenge is to process low-cost, and stable doped nanomaterials with well-controlled properties that can effectively absorb visible light.

Recently, copper has been increasingly investigated as a dopant for titania [12]. Copper oxide is a narrow band gap (cupric oxide, 1.4 eV; cuprous oxide, 2.2 eV) material which has a high-absorption coefficient, but suffers from UV-induced photocorrosion [12]. However, copper oxide coupled with TiO_2 _has been demonstrated to be stable with improved photocatalytic degradation properties [9,13,14], effective CO_2 _photoreduction [15,16], improved gas sensing, and enhanced H_2 _production [17,18]. It has been shown that Cu-doped TiO_2 _induces more toxicity compared to TiO_2 _[19]. Though a large number of studies on Cu-doped TiO_2 _nanomaterials have been reported, there is little information available on the effect of dopant concentration on TiO_2 _properties. Dopants can replace Ti in the substitutional sites or be incorporated in the interstitial sites. In some cases, they may segregate on the surface [20]. The creation of new energy states due to the incorporation of the dopant in the host TiO_2 _alters the particle properties, electronic structure, and light absorption properties. These affect their functionality, and hence can be used in different applications [3,8,20,21]. In summary, there is a need to synthesize Cu-doped nanomaterials with controlled properties (independently) which will help understand in detail the role of the dopant in altering TiO_2 _properties. It is essential to have samples wherein one characteristic is varied, keeping the others the same. For example, samples of varying crystal phases while maintaining the size the same will allow to establish the dependence of biological activity with the crystal phase.

Studies have reported the preparation of various doped TiO_2 _nanomaterials by multi-step liquid-phase synthesis [5], gas-phase spray pyrolysis, and flame synthesis methods [22-24]. Flame aerosol synthesis is a single-step process and allows independent control of the material properties such as particle size, crystallinity, homogeneity, and degree of aggregation [25,26]. At elevated temperatures encountered in the flame synthesis process, most dopants can diffuse rapidly [27] and be uniformly distributed due to excellent precursor vapor mixing at the molecular level [22,20]. Furthermore, flame aerosol processing is a scalable technique that is commercially used to manufacture large quantities of nanomaterials [28].

The synthesis of Cu-doped TiO_2 _in a single-step flame aerosol process is reported in this paper. A detailed characterization of the as-produced samples to understand the influence of process parameters on material properties is done. The role of key process parameters such as molar feed ratio of precursors and dopant concentration on TiO_2 _nanomaterial properties such as size, composition, crystallinity, stability in suspension, and morphology are thoroughly investigated. A method to control the crystal phase of the Cu-doped TiO_2 _nanomaterial has been discussed. The effect of annealing temperature on crystal phase and microstructure of the Cu-doped TiO_2 _material is reported. A formation mechanism of Cu-doped TiO_2 _nanomaterial in the flame aerosol reactor is elucidated.

## Experimental

### Nanomaterial synthesis

**Figure 1:**
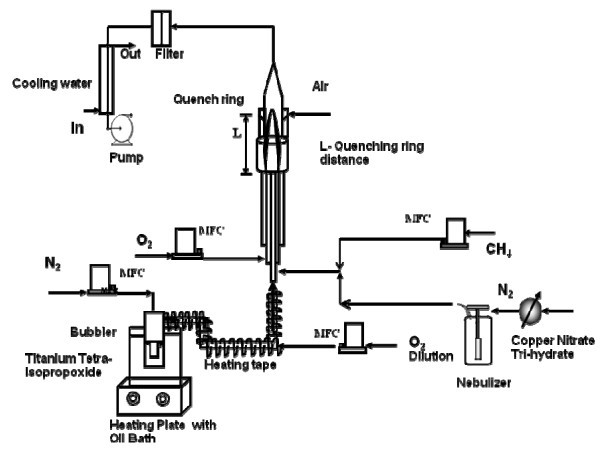
**Schematic diagram of the FLAR experimental setup used to synthesize Cu-doped TiO_2 _nanoparticles**.

Figure 1 shows the schematic diagram of the flame aerosol reactor system used for the synthesis of the Cu-doped TiO_2 _nanomaterials. The main components of the flame aerosol reactor system are: a diffusion burner, a precursor feeding system, and a quenching and collection system. The design details of the diffusion burner used for this study is given in Jiang et al. [26]. Nitrogen was passed through titanium tetra-ispopropoxide (TTIP, 99.7%, Aldrich, Steinheim, Germany) in a bubbler, and the saturated vapor was introduced into the central port of the burner. The bubbler containing the liquid TTIP precursor was placed in an oil bath and was maintained at a temperature of 98°C. The precursor delivery tube was maintained at a temperature of 210°C by a heating tape. This avoided the condensation of the precursor TTIP vapor in the delivery tube. Copper nitrate trihydrate (99.5%, VWR International, Radnor, PA, USA) was used as the dopant precursor. The dopant precursor solution was prepared by dissolving a known amount of copper nitrate in distilled water. A stainless steel collison nebulizer was used to generate fine spray droplets (less than 2 μm), which were then carried by nitrogen gas into the high-temperature zone of the flame. The doping percentage was varied by introducing different molar ratios of both the precursors. The overall doping concentration was varied from 0 to 15 wt.%. Methane and oxygen were introduced into the second and third ports of the burner respectively to create a diffusion flame zone. The volumetric flow rates of N_2 _through the TTIP bubbler and the O_2 _were precisely controlled by mass flow controllers at 2 and 7.5 lpm, respectively. The methane flow rate was maintained at 1.8 lpm, and varied for few of the tests. A 20-lpm flow of compressed air was supplied in a radial direction to the quenching ring for cooling. The entrained air diluted the aerosol stream and suppressed particle growth. The synthesized materials were collected using a glass microfiber filter paper (Whatman) for further characterization.

### Material characterization

The size, morphology, and microstructure of the nanoparticles were determined by a transmission electron microscope (TEM; Model: JEOL 2100F FE-(S) TEM, JEOL Ltd., Tokyo, Japan) with an accelerating voltage of 200 kV and by a field emission scanning electron microscope (SEM) (Model: JEOL 7001LVF FE-SEM, JEOL Ltd.). The elemental analysis of the doped nanomaterial was done using energy dispersive spectroscopy (EDS) analysis integrated with a SEM. Phase structures of the material were determined using an X-ray diffractometer (XRD) with Cu Kα radiation (*λ *= 1.5418 A) (Rigaku D-MAX/A9). Zeta potential, an indicator of the stability of nanoparticles in suspensions, was measured by using a ZetaSizer Nano ZS (Malvern Instruments Ltd., Worcestershire, UK) dynamic light scattering instrument. Nanoparticles were dispersed in de-ionized water at a concentration of 30 μg/ml and sonicated for 25 min using a bath sonicator (40 W, 50 kHz, 5 Fisher Scientific, Fairlawn, New Jersey, USA) before zeta potential measurements. UV-visible absorption spectroscopy (Perkin Elmer Lambda 2S, Perkin Elmer, Waltham, MA, USA) was used to analyze the absorbance spectrum of the nanomaterials over wavelengths ranging from 200 to 800 nm at room temperature. From the absorption spectrum, the band gap was estimated. The absorption edge was estimated to be the point where the absorption was 30% of the maximum, corresponding to where 50% of the photons were absorbed. This approach was used because of the difficulty in finding the linear region of the absorption spectrum according to conventional methods of band gap estimation [21].

### Experimental test plan

The list of experiments performed is outlined in Table 1. The flow rates were controlled to maintain the same residence time in the high-temperature flame (test 1). TiO_2 _was synthesized under the same experimental conditions using only TTIP as the precursor (test 1A). Addition of dopant influences nanomaterial properties such as size, crystal structure, stability in suspension, and optical properties. The copper dopant concentration was varied from 0 to 15 wt.% to process Cu-doped TiO_2 _nanomaterials (test 1(B-F)) to investigate the impact on properties. The copper dopant concentration was estimated based on the precursors feed rate to the flame. The temperature-time history in the flame impacts the particle formation and growth rates. This was varied by altering the methane flow rate from 0.8 to 1.8 lpm at a constant dopant level of 3 wt.% (test 2). Annealing of the 1 and 15-wt.% Cu-doped TiO_2 _was conducted for 4 h at 400 and 600°C in an atmosphere of air to examine property alterations (test 3).

**Table 1:** Summary of the experimental test plan

**Test no**.		Dopant concentration (wt%)	CH_4 _(lpm)	Objective
1	A	0	1.8	Study the influence of dopant concentration on TiO_2 _material properties such as size, crystal phase, suspension stability, and light absorption.
	B	0.5		
	C	1		
	D	3		
	E	5		
	F	15		
2	A	3	0.8	Study the effect of methane flow rate on size and crystal phase of the material.
	B		1.2	
	C		1.5	
	D		1.8	
3	A	1	Annealing temperature, 400°C, 600°C	Examine the effect of annealing on phase and microstructure characteristics of Cu-doped TiO_2 _nanoparticles
	B	15	Duration of annealing under air, 4 h	

All the particles were synthesized by diffusion flame aerosol reactor. Annealing was done in a furnace under air atmosphere.

## Results and discussion

Doping TiO_2 _with other atoms changes properties such as particle size, crystal structure, stability in suspension, and light absorption. The mechanism of Cu-doped TiO_2 _nanoparticle formation in the flame aerosol reactor is discussed first. The effect of copper dopant on TiO_2 _particle properties are discussed followed by crystal structure control of the doped TiO_2 _nanomaterials. Finally, microstructure changes of Cu-doped TiO_2 _are discussed under different annealing conditions.

### Particle formation mechanism

**Figure 2:**
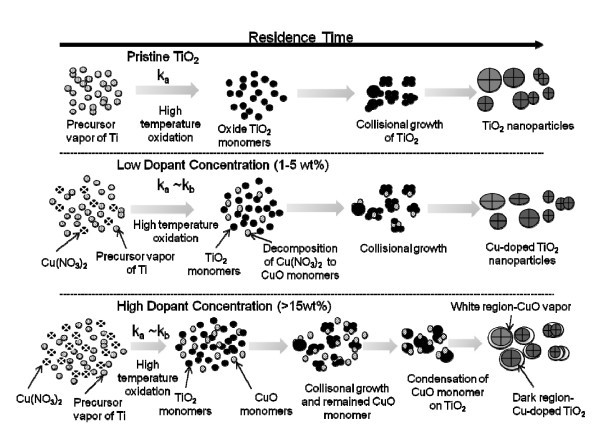
**Cu-doped TiO_2 _nanoparticles formation mechanisms in a FLAR**. Top represents TiO_2 _formation mechanism, middle is for low copper dopant concentration and bottom is for high dopant concentration.

The proposed Cu-doped TiO_2 _particle formation mechanism is illustrated in Figure 2. This is similar to the pathways proposed by Basak [24] for multi-component nanomaterial systems. To understand the formation mechanism of the Cu-doped TiO_2 _nanoparticles in the flame aerosol reactor, pristine TiO_2 _was synthesized first using TTIP only as the precursor. TTIP decomposes to form TiO_2 _monomers, which then undergo subsequent growth by collision followed by sintering to form nanoparticles (test 1A). For synthesizing Cu-doped TiO_2 _particles, both the TTIP and copper nitrate precursor are fed to the high-temperature flame. The nanoparticle properties such as size and composition depend on the relative decomposition kinetics and molar feed ratios of the precursors (see Figure 2). The decomposition rate of TTIP is given by, *k_a _= *3.96 × 10^5 ^exp((-7.05 × 10^4^)/RTs^-1 ^[29]. Since the kinetic data for copper nitrate precursor is not available, the decomposition rate reported for copper acetyl acetonate was assumed (*k_b _= *3.02 × 10^7 ^exp((-1.15 × 10^5^)/RT)s^-1^) [30]. The two precursors form TiO_2 _(formed from TTIP molecular decomposition) and CuO (formed by decomposition of copper nitrate followed by evaporation) monomers at similar time instants as their decomposition rates are similar (*k*_1, Cu _/*k*_1, Ti _to approximately 5, at 2,200°C). Depending on the molar feed ratio of the precursors, a variety of morphologies can be formed, ranging from particles consisting of only copper oxide, particles of only TiO_2_, and the particles of mixed TiO_2 _and CuO. At low copper concentrations (1-5 wt.%), CuO monomers are readily incorporated into the higher concentration TiO_2 _clusters by a scavenging process. This is similar to the phenomenon demonstrated by Wang et al. [22]. Subsequent collisional growth and sintering result in a homogenous mix of Cu-doped TiO_2 _particles. However, at higher Cu feed concentration (approximately 15wt%), apart from the collision and sintering of the CuO monomers and TiO_2 _clusters, some of the CuO oxide monomers also condense onto the formed Cu-doped TiO_2 _particles. The HR-TEM image of the synthesized 15-wt.% Cu-TiO_2 _nanoparticles indicates regions of amorphous CuO on the particle surface. The explanation of CuO monomer condensation on the particle surface is thus corroborated (test 1F). The nanomaterials synthesized at various dopant concentration were verified by single particle EDS analysis to be comprised of both copper and titania. No particles were found consisting of only Ti or only copper species.

### Effect of copper dopant concentration on TiO_2 _properties

#### Particle size analysis

**Figure 3:**
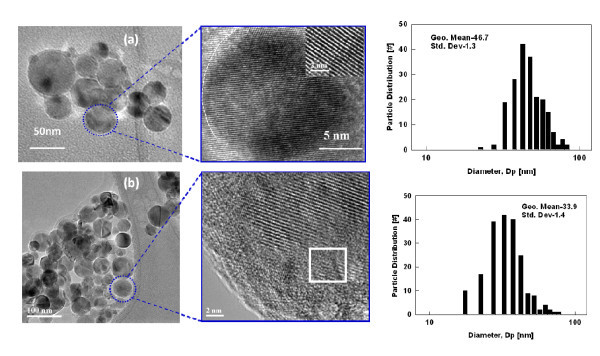
**TEM images and particle size distributions of as synthesized Cu-doped TiO_2 _nanoparticles**. (**a**) 1 wt.% Cu-TiO_2 _and (**b**) 15 wt.% Cu-TiO_2_. Inset is the HR-TEM image of the crystal fringes (test 1). Size distribution of particles is determined from measurement of 200 particles from representative TEM images (test 1B, F).

**Figure 4:**
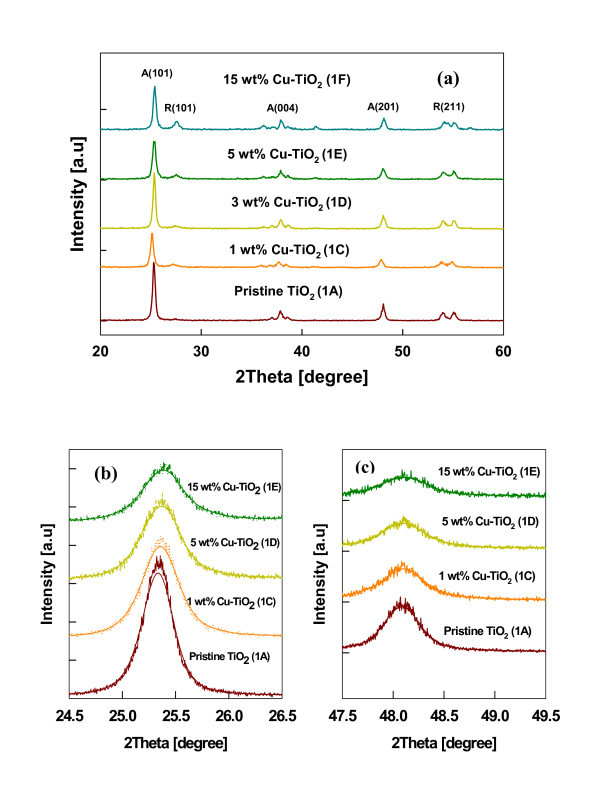
**The XRD diffraction pattern of the Cu-doped TiO_2 _nanomaterials**. (**a**) XRD spectra of as-prepared Cu-TiO_2 _nanoparticles with different dopant concentrations (*A *anatase, *R *rutile). (**b**) Comparison of the XRD anatase peaks of Cu-TiO_2 _nanoparticles: anatase (101) peaks and (**c**) anatase (201) peaks (test 1).

Figure 3 shows the TEM, HR-TEM images, and primary particle size distribution of 1 wt.% Cu-TiO_2 _(test 1B) and 15 wt.% Cu-TiO_2 _(test 1F) samples. The particle size distribution was obtained by measuring the diameter of 200 particles from representative TEM images. As shown in the size distribution of these samples (see Figure 3), the particles were spherical and size decreased with increasing doping concentration. The geometric mean primary particle size obtained at 1 wt.% doping was approximately 47 nm compared to approximately 33 nm obtained at 15 wt.% doping. The peak broadening observed in XRD pattern (see Figure 4) also qualitatively explained the change in particle size and lattice expansion with doping. The crystallite size was estimated from the XRD pattern obtained using Scherrer formula. The crystallite size obtained at 1 wt.% doping was 33 nm compared to 25 and 23 nm at 5 and 15-wt.% doping concentration. It is important to note that crystallite size estimation from XRD is different from the particle size observed from the microscopic analysis. XRD measures the size of the small domains within the grains and one particle may consist of several crystallites based on the preparation methods [31]. The decreased particle size with increasing doping concentration is due to the inhibition of the grain growth. As evident from the HR-TEM images of the 15 wt.% Cu-TiO_2 _(see Figure 3), an enhanced amorphous layer is observed on the surface. The excess CuO monomers condense on to the existing Cu-doped TiO_2 _particles. Thus, particle crystallinity decreases and also prevents grain growth. Wang et al. [22] observed an amorphous crystal structure and decreased grain size with an increasing Fe^2+^/Ti^4+ ^ratios consistent with our Cu-doped TiO_2 _materials. Reduction in size was also observed when Li et al. [3] synthesized Zn-doped SnO_2 _nanomaterials. Norris et al. [27] proposed a process called self-purification by which dopants diffuse from inside to the surface sites of TiO_2 _nanocrystals. This change in particle size with doping concentration is fundamentally a very important phenomenon for electronic structure modification. These results indicate that the particle size of the Cu-doped TiO_2 _can be controlled by manipulating the dopant concentration in addition to the methods demonstrated by other researchers by controlling the precursor feed concentration and residence time of the particle in the high-temperature flame [26,32].

#### Crystal phase

The functionality of TiO_2 _nanomaterials for various applications depends on its crystal phase. The anatase phase of TiO_2 _is preferred for photocataytic applications, whereas rutile phase is preferred for applications in pigments [1]. It is, therefore, necessary to understand the modifications in the crystal structure by incorporation of the dopants in TiO_2_. The XRD diffraction pattern of the Cu-doped TiO_2 _nanomaterials synthesized at various concentrations is shown in Figure 4. The pristine and Cu-doped TiO_2 _nanoparticles were prepared at the same flame conditions for comparison. The pristine TiO_2 _was primarily anatase under the chosen processing conditions. However, with increasing dopant concentration, the transformation from anatase to rutile phase occurred, as shown in Figure 4a from the (110) rutile peak, consistent with other studies [18,33]. The anatase and rutile fraction were calculated according to the formula proposed by Spurr and Myers [34]. The pristine TiO_2 _had 1.2% rutile content, but with increasing doping concentration to 15 wt.%, the rutile phase increased to 21.8%. Even at high dopant concentration (15 wt.%), no pure dopant-related crystal phase was observed within the XRD detection limit. The same anatase to rutile phase transformation was observed for synthesis of Cu-doped TiO_2 _by other methods [9,35].

The similarity in ionic radius of Cu^2+ ^(0.73 Å) to that of Ti^4+ ^(0.64 Å) enable copper to substitutionally replaces Ti in the titanium lattice in the flame environment, where particles are formed from the atomistic state. In the high-temperature flame synthesis of Cu-doped TiO2 nanomaterial, the copper dopant creates a higher number of defects inside the anatase phase, resulting in a faster formation and growth of a higher number of rutile nuclei [36]. At elevated temperatures, the substitution of Ti^4+ ^by Cu^2+ ^increases the oxygen vacancy concentration and decreases the free electron concentration. The excess of oxygen vacancies created in the TiO_2 _crystal lattice is the responsible for anatase to rutile phase transition [36,37]. Nair et al. [36] found that a dopant with an oxidation state above 4+ will reduce the oxygen vacancy concentration in the titania lattice as an interstitial impurity. Dopants with an oxidation state of 3+ or lower when placed in the titania lattice points create a charge-compensating anion vacancy [36] and cause a transformation to the rutile phase as also found in this study. At higher dopant concentration (15 wt.%) amorphous phase was also observed on the surface as well as in the bulk. The TEM and HR-TEM images 1 and 15-wt.% Cu-doped TiO_2 _nanoparticles (see Figure 3) shows that particles at lower doping concentrations are fully crystallized, and the crystal lattice spacing corresponds to the anatase phase of TiO_2 _(0.331 ± 0.03 nm), whereas the particle synthesized at 15-wt.% copper concentration shows both crystalline and amorphous phases of the material. The HR-TEM images confirm that Cu^2+ ^doping retards the grain growth of TiO_2 _nanoparticles. Similar results of decreasing crystalline nature of material were observed when Fe^2+^- and Zn^2+^-doped TiO_2 _were synthesized [3,22]. In a similar doping study, Wang et al. [22] found that at higher Fe^2+^/Ti^4+ ^ratios of 0.12, more rutile and amorphous crystal structure was observed, consistent with our Cu-doped TiO_2 _materials.

Figure 4b and 4c represent the XRD spectra for (101) and (201) anatase peaks scanned at a very small steps of 0.004 degree for pristine and doped TiO_2 _nanomaterials. It is important to note that with increasing dopant concentration, broadening of the major anatase peaks (101) and (201) was observed, which indicates a decrease in crystallite size. The shift in peak position to the right [8] with increasing dopant concentration indicates that Cu^2+ ^ions replaced some Ti^4+ ^ions along with the lattice expansion. The results clearly indicate that addition of dopant alters the crystal phase of the host nanomaterial and the degree of phase transition depends on dopant types and their concentrations.

#### Zeta potential and suspension stability

**Figure 5:**
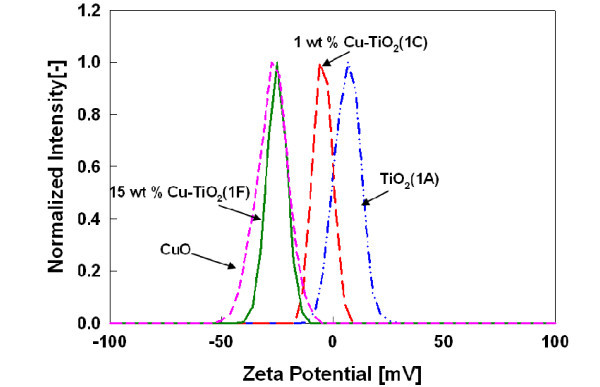
**Zeta potential measurements of Cu-doped TiO_2 _nanoparticles in aqueous suspension**.

The dispersion characteristics of nanoparticles in aqueous suspensions influence the fate and transport, catalytic reactivity in the environmental system as well as critical in understanding for toxicological applications [38,39]. The stability of the synthesized Cu-doped TiO_2 _nanoparticles was analyzed through the measurement of zeta potential in aqueous system using de-ionized water suspension (Figure 5) and compared with pure TiO_2 _(test 1A) and commercial CuO. When metal oxide nanoparticles are dispersed in water, the hydration of the nanoparticle surface followed by protonation and deprotonation of the surface groups from the oxide surface results in a surface charge. The effective surface charge on the particle depends on the isoelectric point (IEP) in the suspension [39,40]. The zeta potential observed for pure TiO_2 _particle was +3.4 mV in the suspension, as the measured pH of the suspension was 5.06, which is less than the IEP of the TiO_2_(pH approximately 6.0) and consistent with other studies [40]. However, for Cu-doped TiO2 nanoparticles, the zeta potential value decreased to -3.4 mV and -25.6 mV at 1-wt.% (test 1B) and 15-wt.% (test 1F) copper dopant concentration. The zeta potential measured for the commercial CuO was -27.3 mV which is close to the zeta potential value observed for 15-wt.% Cu-TiO_2 _samples (test 1F). The high surface charge on the 15 wt.% Cu-TiO_2 _indicates better stability of these particles over pristine TiO_2 _nanoparticles in aqueous suspension. The higher zeta potential value and suspension stability of the doped nanoparticles compared to TiO_2 _is attributed to charge imbalance created due to substitution of Ti^4+ ^atoms by Cu^2+ ^in the TiO_2 _structure resulting in a more negatively charged surface. Furthermore, zeta potential values for 15-wt.% Cu-TiO_2 _samples being similar to pure CuO supports the presence of a copper oxide layer on the outer surface of the particles.

#### Light absorption properties

**Figure 6:**
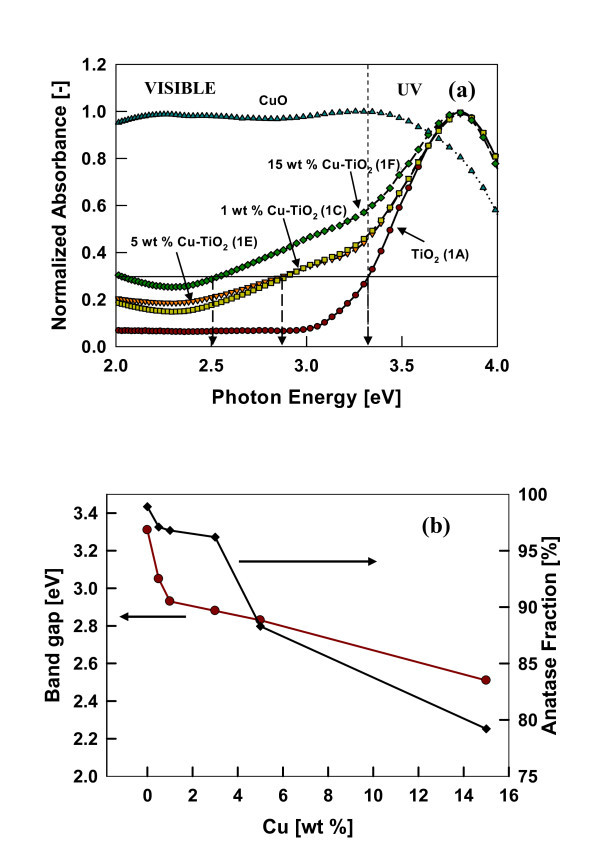
**Absorption spectrum of Cu-doped TiO_2 _nanomaterials prepared at various dopant concentrations**. (**a**) Normalized UV-visible absorption spectra measured by diffuse reflectance spectroscopy. (**b**) Estimated band gap as a function of dopant concentrations (test 1)

The absorption spectra of the resulting Cu-doped TiO_2 _nanomaterials was determined by a diffusive reflectance spectroscopy measurement. The absorption spectrum of Cu-doped TiO_2 _nanomaterials prepared at various dopant concentrations are shown in Figure 6. With increasing dopant concentration, an increased absorbance in the visible spectrum is observed. The estimated Eg for pristine TiO_2 _was 3.31 eV which is consistent with the reported value for anatase TiO_2 _[21]. With increasing dopant concentration, the band gap energy decreased and was estimated to be 2.51 eV at the highest dopant concentration of 15 wt.%. This change of approximately 0.8 eV was due to the incorporation of Cu^2+ ^ions into TiO_2 _crystal structure, and CuO forming a layer on the particle surface. From an experimental and theoretical study of band structure estimation of metal oxides, The results are consistent with findings of Thimsen et al. [21] that the band gap energy decreases with increasing Fe concentration in anatase-based TiO_2 _materials.

Change in the optical absorption is due to the defect centers created by the substitution of Ti^4+ ^by Cu^2+ ^atoms in the TiO_2 _crystal lattice. Earlier studies indicated that doping with aliovalent ions changes the local lattice symmetry and defect characteristics, which could change the absorption properties and the material properties. In Cu-dopedTiO_2_, when copper ions are either located inside the bulk TiO_2 _or on the surface sites, a rearrangement of the neighbor atoms take place to compensate the charge deficiency, resulting in lattice deformation. The lattice deformation affects the electronic structure causing the band gap shift [3]. Furthermore, small amounts of Cu^2+ ^dopant in the lattice sites of TiO_2 _introduce oxygen vacancies due to the charge compensation effect [36,41]. Increasing the copper doping concentration increases the oxygen vacancies and probably form a newly doubly occupied oxygen vacancy as discussed in Li et al. [3]. Therefore absorption of the doped nanomaterial and band gap shift may be controlled by surface effects, doping-induced vacancies, and lattice strain. It can be said that the copper modified TiO_2 _structure extends its absorption to the visible spectrum of sunlight (400-700 nm) effectively. Hence, these copper-doped materials can be utilized for various visible-light photocatalytic applications, which have been demonstrated in several other studies [9,18].

#### Crystal phase control of Cu-doped TiO_2 _nanoparticle

**Figure 7:**
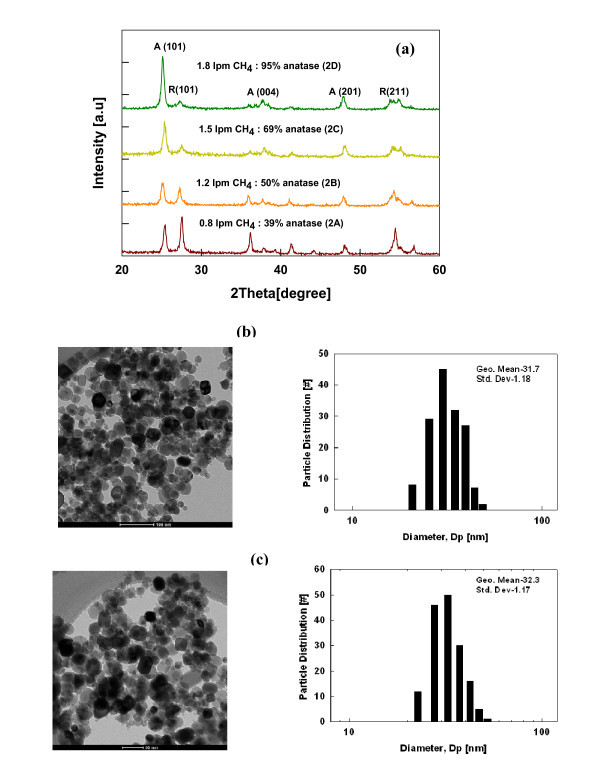
**Dopant concentration, representative TEM micrographs and corresponding size distribution of the particles**. (**a**) XRD spectra at different methane flow rates (*A *anatase, *R *rutile) and particle size distributions at (**b**) 0.8 lpm, (**c**) 1.2 lpm methane flow rates for 3-wt.% Cu-TiO_2 _nanoparticles (test 2).

The functionality of the nanomaterials depends on their properties such as particle size, crystal phase, morphology, and agglomeration [38,40]. A recent study by Braydich-Stolle et al. [42] showed that cytotoxicity in the cells is both size and crystal structure dependent. They demonstrated that mechanism of cell death varied with different crystal structure; the anatase phase of TiO_2 _being more toxic than the rutile phase. To understand the role of crystal phase of the doped nanomaterials on its functionality, it is important to independently control the crystal phase without varying the other material properties such as size. Previous studies have demonstrated that crystal phase of the TiO_2 _nanoparticle can be controlled by varying the temperature in the flame (changing the methane flow rates) and quenching rate downstream of the flame [25,26]. A similar methodology was adopted to control the crystal phase of the Cu-doped TiO_2 _materials. The dopant concentration was kept constant at 3 wt.% and methane flow was varied from 0.8 to 1.8 lpm (test 2, Figure 7a). The anatase phase varied from 39% to 95%, when the methane flow was increased from 0.8 to 1.2 lpm, whereas the primary particle sizes for all the cases were similar. The representative TEM micrographs and corresponding size distribution of the particles synthesized at 0.8 and 1.8 lpm are shown in Figure 7b, c. The geometric mean size of 31.5 and 32.3 nm were nearly the same for the two flow rate conditions. The size remained similar due to the balance between temperature profile and residence time in the flame at different methane flow rates. For a fixed flame operating parameters, increasing the methane flow rate increases the flame temperature but at the same time reduces the residence time in the flame. For lower methane flow rate the temperature decreases and residence time increases. Thus the crystal phase of the Cu-doped TiO_2 _nanoparticles was independently varied while keeping the primary particle size the same. These well-controlled Cu-doped TiO_2 _samples will be of significant importance in biological studies to elucidate the role of crystal phases without interferences from the other particle properties such as size.

#### Effect of annealing on Cu-doped TiO_2 _nanoparticle properties

**Figure 8:**
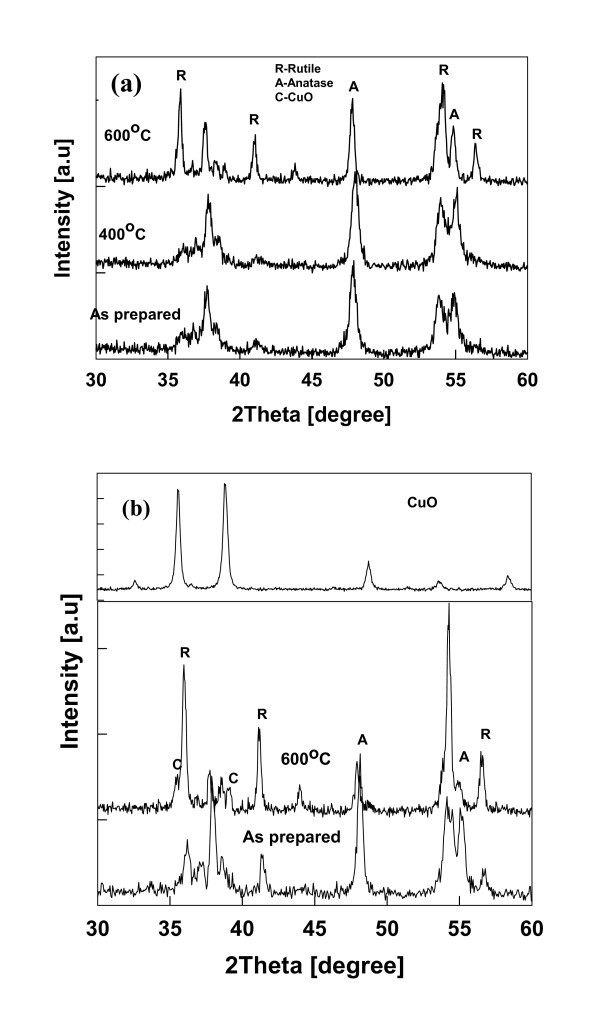
**XRD pattern of the annealed Cu-TiO_2 _nanoparticles**. (**a**) 1-wt.% Cu-TiO_2 _(**b**) 15-wt.% Cu-TiO_2_. *A *anatase, *R *rutile. Samples were annealed for 4 h in a furnace at constant temperature (test 3).

The morphological and structural transformation of the doped nanoparticles plays important role in photocatalytic activity by modifying the surface chemistry, crystal and electronic structure [43]. Since both amorphous and crystalline phases were observed in HR-TEM images at higher dopant concentration, the as-prepared Cu-doped TiO_2 _samples were annealed at different temperatures to investigate the effect on crystal structure and morphology. The 1 and 15-wt.% Cu-doped TiO_2 _samples were annealed at temperatures of 400°C and 600°C for 6 h. No phase transformation was observed at 400°C. At 600°C, the transformation from anatase to rutile phase was observed as shown in Figure 8, which is consistent with other studies [18,44]. The anatase weight fraction decreased from 75% to 21% for the 15-wt.% Cu-doped TiO_2 _sample. However, the morphology of the particles changed from spherical to hexagonal structure for nanoparticles prepared at both the dopant concentrations. The crystallite size increased with annealing. For 15-wt.% Cu-doped TiO_2 _sample, the phase related to CuO was observed based on the peaks recorded at Bragg angle of 35.5 and 39 from the XRD pattern (Figure 8). The amorphous CuO present in the outer layers were annealed to form the crystalline phase in the presence of air.

**Figure 9:**
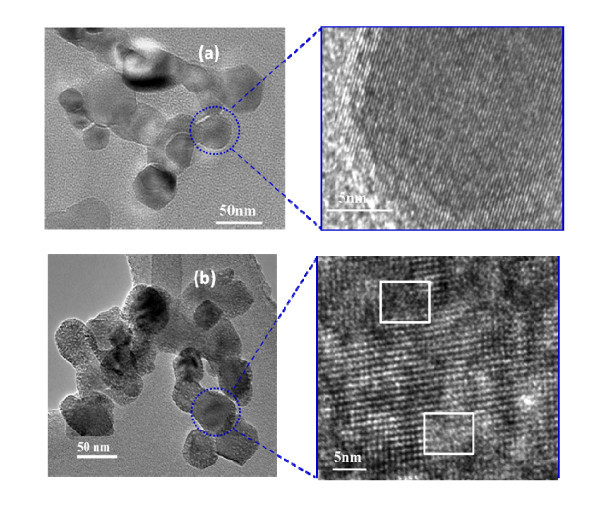
**TEM images of annealed Cu-doped TiO_2 _samples**. (**a**) 1 wt.% Cu-TiO_2 _and (**b**) 15 wt.% Cu-TiO_2_. Annealing temperature, 600°C; duration of annealing, 4 h (test 3).

**Figure 10:**
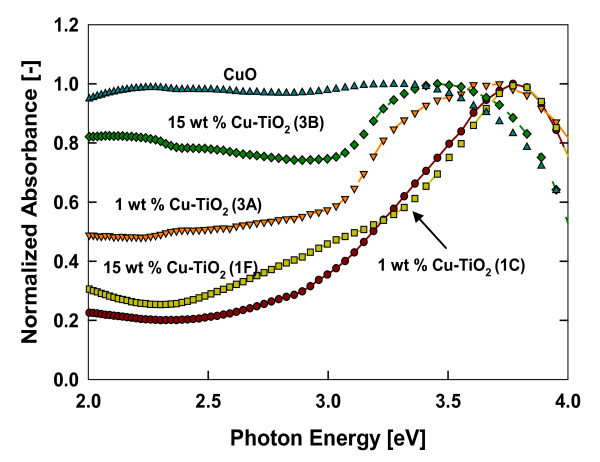
**Normalized UV-visible absorption spectra measured by diffuse reflectance spectroscopy of the annealed Cu-doped TiO_2 _nanomaterials**. Samples were annealed for 4 h at 600°C (test 3).

The HR-TEM images of samples annealed at 600°C are shown in Figure 9. The figure indicates that the annealed 1-wt.% Cu-doped TiO_2 _particle was completely crystallized with no discontinuity in the crystal fringes as observed from HR-TEM images, similar to the as-prepared 1-wt.% Cu-doped TiO_2 _particles. However, for the 15-wt.% dopant sample, some amorphous regions were still detected as shown in Figure 9 (highlighted with the white squares). More detailed investigations are needed to understand the effect of dopant concentration and reaction environments on morphology change during post-synthesis treatment of the initially synthesized spherical particles. The UV-vis measurements of absorption spectra of 1- and 15-wt.% Cu-doped TiO_2 _annealed samples are shown in Figure 10 and compared with the commercially available CuO nanoparticles. Annealing of the 15 wt.% Cu-TiO_2 _increased the absorption compared to the as prepared samples in the visible spectrum mainly because of enhanced crystalline CuO formation. It is clear from the results that post-synthesis annealing can alter the doped TiO_2 _nanomaterial properties such as size, crystal structures as well as absorption properties, thus influencing eventual functionality and performance.

## Conclusions

Cu-doped TiO_2 _nanoparticles were synthesized in a diffusion flame aerosol reactor and the properties were readily varied by controlling the processing conditions. The increase in dopant concentration caused the transformation from anatase to rutile phase of TiO_2 _due to replacement of Ti^4+ ^by Cu^2+ ^in the crystal structure of TiO_2_. A decrease in primary particle size was also observed. The doped nanomaterials exhibited better aqueous suspension stability compared to pristine TiO_2 _due to charge imbalance created. The annealing of the doped samples resulted in the phase segregation and crystallization of CuO for the higher dopant concentration samples. Spectroscopy measurements confirm a shift in the absorption to visible frequencies, due to crystal structure modification.

## Competing interests

The authors declare that they have no competing interests.

## Authors' contributions

PB and MS both participated in the design of the study. MS carried out all the experiments. MS and PB participated in results analysis. MS drafted the manuscript and PB provided comments/suggestions to revise it.
